# Factors associated with involuntary psychiatric hospitalization in Portugal

**DOI:** 10.1186/s13033-021-00460-4

**Published:** 2021-04-20

**Authors:** Manuela Silva, Ana Antunes, Sofia Azeredo-Lopes, Adriana Loureiro, Benedetto Saraceno, José Miguel Caldas-de-Almeida, Graça Cardoso

**Affiliations:** 1 Comprehensive Health Research Centre (CHRC), NOVA Medical School, NMS, Universidade Nova de Lisboa, Lisbon, Portugal; 2 Lisbon Institute of Global Mental Health, Lisbon, Portugal; 3 NOVA Medical School, NMS, Universidade Nova de Lisboa, Lisbon, Portugal; 4 Centre of Studies on Geography and Spatial Planning (CEGOT), Faculty of Arts and Humanities, Coimbra, Portugal

**Keywords:** Mental health services, Involuntary psychiatric treatment, Involuntary psychiatric hospitalization, Compulsory admission, Health policy, Health system

## Abstract

**Background:**

Identifying which factors contribute to involuntary psychiatric hospitalization may support initiatives to reduce its frequency. This study examines the sociodemographic, clinical, and contextual factors associated with involuntary hospitalization of patients from five Portuguese psychiatric departments in 2002, 2007 and 2012.

**Methods:**

Data from all admissions were extracted from clinical files. A Poisson generalized linear model estimated the association between the number of involuntary hospitalizations per patient in one year and sociodemographic, clinical, and contextual factors.

**Results:**

An increment of involuntary hospitalizations was associated with male gender [exp($\widehat{\upbeta }$) = 1.31; 95%CI 1.06–1.62, p < 0.05], having secondary and higher education [exp($\widehat{\upbeta }$) = 1.45; 95%CI 1.05–2.01, p < 0.05, and exp($\widehat{\upbeta }$) = 1.89; 95%CI 1.38–2.60, p < 0.001, respectively], a psychiatric diagnosis of psychosis [exp($\widehat{\upbeta }$) = 2.02; 95%CI 1.59–2.59, p < 0.001], and being admitted in 2007 and in 2012 [exp($\widehat{\upbeta }$) = 1.61; 95%CI 1.21–2.16, p < 0.01, and exp($\widehat{\upbeta }$) = 1.73; 95%CI 1.31–2.32, p < 0.001, respectively]. A decrease in involuntary hospitalizations was associated with being married/cohabitating [exp($\widehat{\upbeta }$) = 0.74; 95%CI 0.56–0.99, p < 0.05], having experienced a suicide attempt [exp($\widehat{\upbeta }$) = 0.26; 95%CI 0.15–0.42, p < 0.001], and belonging to the catchment area of three of the psychiatric services evaluated [exp($\widehat{\upbeta }$) = 0.65; 95%CI 0.49–0.86, p < 0.01, exp($\widehat{\upbeta }$) = 0.67; 95%CI 0.49–0.90, p < 0.01, and exp($\widehat{\upbeta }$) = 0.67; 95%CI 0.46–0.96, p < 0.05 for Hospital de Magalhães Lemos, Centro Hospitalar Psiquiátrico de Lisboa and Unidade Local de Saúde do Baixo Alentejo, respectively].

**Conclusions:**

The findings suggest that involuntary psychiatric hospitalizations in Portugal are associated with several sociodemographic, clinical, and contextual factors. This information may help identify high-risk patients and inform the development of better-targeted preventive interventions to reduce these hospitalizations.

## Background

The use of involuntary hospitalization of people with mental disorders is a central and controversial issue in mental health care. For more than one hundred years, there has been a debate on how to balance different and often contradictory interests, such as the principle of personal freedom and basic human rights, the need for adequate treatment, and public safety [1, 2]. Involuntary hospitalization is now seen as the way to achieve the highest attainable standard of health when a severe exacerbation of illness impairs decision-making capacity [3], and can be lifesaving [4]. However, it represents a deprivation of personal liberty and a suspension of legal capacity [5], and conflicts with the right to personal autonomy and to make decisions about one’s own treatment [6]. Existing observational studies suggest that involuntarily admitted patients show limited clinical and social improvement [7–11], with mixed evidence on the impact on suicidality [11, 12]. At follow-ups, many of the patients view their admission and treatment positively [7, 8, 13, 14], but a substantial percentage of them retrospectively do not feel that the admission was justified and beneficial [7, 13]. Empirical data suggest that involuntary hospitalization may be experienced as traumatic and stigmatizing [15], lead to low levels of treatment satisfaction [4, 16], have negative effects on patient–therapist relationship [17], lead to long-term avoidance of mental health care [4, 15, 18], and increase the risk of emergency compulsory re-hospitalization [19] and further coercive measures during the hospital stay [6, 20, 21]. The United Nations (UN) Convention on the Rights of Persons with Disabilities (CRPD), the most up-to-date international legal instrument specifically tailored to stipulate the rights of persons with disabilities [22–24], sparked a global debate [14, 23, 25–28] by considering that all persons have legal capacity at all times, irrespective of mental status, and that substitute decision-making and involuntary hospitalization are indefensible [3, 23, 26, 29].

A central objective of legal frameworks for involuntary hospitalizations and their subsequent revisions was to minimize them [2, 30, 31]. However, rates of involuntary hospitalization have varied strikingly across and within countries in the past three decades [2, 32–34], with rates increasing over time in many countries [4, 19, 30, 35, 36]. The factors influencing involuntary hospitalization have been classified as: (1) individual-related factors, including the sociodemographic and clinical features of the affected persons and the attitudes and clinical competence of their caregivers; (2) system-related factors, including the organization and resources of mental health care; and (3) area-related factors, including the national legislation, the wider societal perspective and traditions, socioeconomic factors, and economic changes [37, 38]. The few data available on these risk factors are often controversial and difficult to interpret. Further research in this area is warranted [38].

A systematic review, meta-analysis, and narrative synthesis of 77 studies from 22 countries found that the factors most strongly associated with involuntary psychiatric hospitalization are a diagnosis of a psychotic disorder and a previous involuntary hospitalization [4]. On a population level, a positive dose–response relation was found between area-level deprivation and increased rates of involuntary hospitalization [4]. Meta-analysis results also identified male gender, single marital status, unemployment, being in receipt of welfare benefits, and not owning one’s own home as risk factors for involuntary admissions [4]. Using narrative synthesis, the factors found to influence involuntary admissions were positive symptoms of psychosis, perceived risk to others, clinician-rated lack of insight, lack of adherence to treatment before hospitalization, scant social support, and police (vs. family doctor) involvement in admission [4].

In Portugal, the 1998 Mental Health Act establishes the rights of people who are mentally ill and the principles that govern their compulsory detention [39, 40]. This Act is currently under review to fully comply with the twin objectives of reducing coercive measures and enhancing patient autonomy. Portugal has relatively low annual rates of involuntary hospitalization (6 per 100,000 individuals in 2000 and 18.19 per 100,000 individuals in 2013) [32, 34], but few national data are available. To our knowledge, evidence on the risk factors for involuntary psychiatric hospitalization in Portugal is scarce or non-existent. The purpose of this study is to identify sociodemographic, clinical, and contextual factors associated with a high risk of involuntary psychiatric hospitalization of adults in Portugal. The identification of these factors could help better identify high-risk patients, develop more precise preventive interventions to reduce these hospitalizations, and ultimately develop less restrictive and less coercive alternatives.

## Methods

### Design and study sample

This study was part of the research project “Mental Health, Impact Assessment of Local and Economic Constraints—SMAILE”, funded by the Foundation for Science and Technology (PTDC/ATP-GEO/4101/2012). This retrospective cross-sectional study is based on a detailed analysis of all inpatient mental health records from five adult public psychiatric departments during 2002, 2007 and 2012. The objective of this study was to assess the use of mental health services in times of economic crisis. Consequently, the years were selected to represent periods before the Great Recession (2002 and 2007) and the period of economic crisis (2012). The data of interest was extracted from patient clinical files in a systematic manner. Inpatients for electroconvulsive therapy were excluded. All other hospitalizations in the three years were included. The study was conducted in psychiatric departments in the Metropolitan Areas of Lisboa and Porto, and the region of Baixo Alentejo, described in Table 1. They were selected for the purpose of covering municipalities with distinct geographical and socioeconomic characteristics, and included consolidated urban areas (Lisboa and Porto), recent urban growth areas with low socioeconomic status characteristics (Amadora), recent urban growth areas with high socioeconomic status characteristics (Oeiras, Póvoa de Varzim and Vila do Conde), and rural areas (Aljustrel, Almodôvar, Alvito, Barrancos, Beja, Castro Verde, Cuba, Ferreira do Alentejo, Mafra, Mértola, Moura, Ourique, Serpa, and Vidigueira). Patients living in the catchment area of each hospital were admitted to the respective department, with the exception of Unidade Local de Saúde do Baixo Alentejo EPE, which had no acute inpatient service, and whose patients were admitted to Centro Hospitalar Psiquiátrico de Lisboa (180 kms away) after evaluation in the local emergency department. The psychiatric departments where the study was conducted are quite different from each other and underwent significant changes between 2002 and 2012, as mental health reform was underway in the country. Two of the hospitals (Centro Hospitalar Psiquiátrico de Lisboa and Hospital de Magalhães Lemos EPE) are big psychiatric hospitals with a pavilion organization and large catchment areas [41], and suffered an important reduction in the number of acute beds during the period under study (Centro Hospitalar Psiquiátrico de Lisboa: 301 in 2005 and 134 in 2012; Hospital de Magalhães Lemos, EPE: 142 beds in 2005 and 99 in 2012) [42, 43]. Two other hospitals (Centro Hospitalar de Lisboa Ocidental EPE and Hospital Professor Doutor Fernando Fonseca EPE) have multidisciplinary community teams, belong to general hospitals [41], and experienced fewer significant changes during the study period. The Unidade Local de Saúde do Baixo Alentejo EPE also belongs to a general hospital, covers a large geographical area, and had no acute inpatient service.

**Table 1:** Characterization of the study areas and psychiatric departments

	Hospital Professor Doutor Fernando Fonseca EPE	Centro Hospitalar de Lisboa Ocidental EPE	Centro Hospitalar Psiquiátrico de Lisboa	Hospital de Magalhães Lemos EPE	Unidade Local de Saúde do Baixo Alentejo EPE
**Characteristics of the hospital**	General hospital with community teams	General hospital with community teams	Psychiatric Hospital	Psychiatric Hospital	General hospital
**Study areas (municipalities)**	Amadora	Lisboa (Western parishes) and Oeiras	Lisboa (Eastern parishes) and Mafra	Porto, Póvoa de Varzim and Vila do Conde	Aljustrel, Almodôvar, Alvito, Barrancos, Beja, Castro Verde, Cuba, Ferreira do Alentejo, Mértola, Moura, Ourique, Serpa, Vidigueira
**Resident population in the study areas (inhabitants)**					
2001	175,872	212,386	199,160	284,971	135,105
2011	175,136	218,208	213,863	279,310	126,692
**Population growth between 2001 and 2011 (%)**	− 0.4	2.7	7.4	− 2.0	− 6.2
**Population density (inhabitant/Km**^**2**^**)**					
2001	7551	3613	792	1121	16
2011	7368	3704	848	1098	15
**Ageing index (individuals aged 65 or older per 100 aged 0 to 14 years)**					
2001	94	132	173	97	176
2011	126	142	151	128	189
**Unemployment rate (%)**					
2001	7.7	6.4	5.6	6.4	12.1
2011	14.9	10.8	10.7	14.4	15.1
**Population with higher education (%)**					
2001	12.0	19.4	10.3	7.2	3.0
2011	17.9	32.8	19.3	13.5	6.2
**One person household (%)**					
2001	21.2	23.4	21.7	13.7	22.4
2011	27.7	29	24.7	17.3	26.6
**Average monthly earnings (€)**					
2004	1045.1	1405.3	1016.8	821.1	716.6
2011	1249.4	1648.8	1232.7	1049.5	900.7

Sources: Statistics Portugal Censos 2001 and 2011; Strategy and Planning Office of the Ministry of Labour, Solidarity and Social Security

The ethics committee of each hospital approved the research, and confidentiality of all information gathered was ensured.

### Measurements

#### Dependent variable

The dependent variable was the number of involuntary psychiatric hospitalizations per patient in 1 year.

#### Independent variables

The independent variables included the individual characteristics of the participants, the year of admission, and the psychiatric service.

For each admission, we extracted information on patient sociodemographic and clinical characteristics, such as age, gender, marital status, education, employment status, psychiatric diagnosis, and presence of a suicide attempt in the last 12 months. Age was grouped into four categories (15–29; 30–49; 50–64; ≥ 65 years). Marital status was categorized into three groups (single; married or cohabitating; divorced, separated or widowed). Education was divided into four categories [none or primary education (≤ 4 years); basic education (5–9 years); secondary education (10–12 years); and higher education (> 12 years)]. Employment status was assessed into three categories [workers (including on sick leave) or students; unemployed; retired or other (including homemakers)].

Psychiatric main diagnoses were established according to the criteria of the International Classification of Diseases, 9th revision, the clinical coding criteria used in Portugal throughout the period of time of this study. They were categorized into five groups: mood and anxiety disorders; dementia; substance use disorders; psychosis; and other mental disorders.

The years of evaluation were 2002, 2007, and 2012.

The data were retrieved from the clinical records of the abovementioned hospitals. The clinical records of the patients from Unidade Local de Saúde do Baixo Alentejo EPE were obtained from Centro Hospitalar Psiquiátrico de Lisboa, where they were admitted.

### Statistical analysis

Descriptive statistics were performed using frequencies and percentages.

A Poisson generalized linear model (GLM) was employed for modelling the expected number of involuntary hospitalizations as a function of the following covariates: gender, age group, marital status, education, employment status, suicide attempt, psychiatric diagnosis, year of evaluation and psychiatric service. The amount of missing data was not relevant and missing data were not handled. Overdispersion was not present as the data did not exhibit greater variation than was expected for this model. The statistical test to check for overdispersion in this Poisson GLM provided a p-value equal to 0.7. The goodness-of-fit of the model was assessed using the deviance of 1347.4 on 2248 degrees of freedom which, with a Chi-Square distribution, gives a clear indication that the model fits the data (p > 0.995).

The R statistical software [44] was used to perform all the statistical analyses.

## Results

### Descriptive statistics

Table 2 shows the number of involuntary hospitalizations in the study sample. Of the 3871 participants, 16.2% (n = 604) had at least one involuntary hospitalization in the previous year. Of these, 90.6% (n = 547) had one involuntary hospitalization, 7.8% (n = 47) had two hospitalizations, 1.2% (n = 7) had three hospitalizations, and 0.5% (n = 3) had four hospitalizations.

**Table 2:** Frequency of involuntary hospitalizations in the study sample

Number of involuntary hospitalizations									
0		1		2		3		4	
n	%	n	%	n	%	n	%	n	%
3127	83.8	547	14.7	47	1.3	7	0.2	3	0.1

Table 3 shows the sociodemographic, clinical, and contextual characteristics of the study sample and the sub-sample with at least one involuntary hospitalization.

**Table 3:** Sociodemographic, clinical, and contextual characteristics of the study sample and sub-sample with at least one involuntary hospitalization

	Full sample (n = 3871)		Respondents with ≥ 1 involuntary hospitalization (n = 604)	
	n	%	n	%
**Sociodemographic characteristics**				
Gender				
Women	1977	51.1	249	41.2
Men	1894	48.9	355	58.8
Age				
15–29	679	17.5	112	18.5
30–49	1802	46.5	317	52.5
50–64	826	21.3	117	19.4
≥ 65	565	14.6	58	9.6
Marital status				
Single	1702	45.5	356	61.0
Married/cohabitating	1222	32.6	113	19.3
Divorced/separated/widowed	819	21.9	115	19.7
Education				
None or primary education	773	31.9	84	21.3
Basic education	858	35.4	143	36.2
Secondary education	404	16.7	77	19.5
Higher education	390	16.1	91	23.0
Employment status				
Workers or students	1076	31.3	165	29.4
Unemployed	915	26.6	180	32.0
Retired or others	1445	42.1	217	38.6
**Clinical characteristics**				
Psychiatric diagnosis				
Mood and anxiety disorders	1603	41.7	154	25.6
Dementia	204	5.3	15	2.5
Substance use disorders	335	8.7	45	7.5
Psychosis	1269	33.0	338	56.1
Other mental disorders	433	11.3	50	8.3
Suicide attempt				
Yes	610	16.4	51	8.6
No	3117	83.6	545	91.4
**Contextual characteristics**				
Year				
2002	1188	30.7	115	19.0
2007	1309	33.8	226	37.4
2012	1375	35.5	263	43.5
Psychiatric service				
Centro Hospitalar de Lisboa Ocidental EPE	523	13.5	138	22.8
Hospital de Magalhães Lemos EPE	1556	40.2	177	29.3
Centro Hospitalar Psiquiátrico de Lisboa	991	25.6	138	22.8
Hospital Professor Doutor Fernando Fonseca EPE	462	11.9	88	14.6
Unidade Local de Saúde do Baixo Alentejo EPE	340	8.8	63	10.4

### Association between involuntary hospitalization(s) and sociodemographic, clinical, and contextual variables

The results of the multivariable Poisson regression model are presented in Table 4. We found that the following factors are independently associated with involuntary hospitalizations: gender, marital status, education, psychiatric diagnosis, a previous suicide attempt, year of admission, and psychiatric service.

**Table 4:** Multivariable Poisson regression model of the association between the number of involuntary hospitalizations and sociodemographic, clinical, and contextual characteristics

	Exp ($\widehat{\upbeta }$)	95%CI
**Sociodemographic characteristics**		
Gender		
Women	Ref	
Men	1.31	1.06–1.62 *
Age		
15–29	Ref	
30–49	1.10	0.86–1.42
50–64	0.97	0.69–1.38
≥ 65	0.89	0.52–1.49
Marital status		
Single	Ref	
Married/cohabitating	0.74	0.56–0.99 *
Divorced/separated/widowed	0.94	0.70–1.24
Education		
None or primary education	Ref	
Basic education	1.30	0.98–1.73
Secondary education	1.45	1.05–2.01 *
Higher education	1.89	1.38–2.60 ***
Employment status		
Workers or students	Ref	
Unemployed	1.08	0.84–1.39
Retired or others	1.11	0.86–1.45
**Clinical characteristics**		
Psychiatric diagnosis		
Mood and anxiety disorders	Ref	
Dementia	0.98	0.46–1.92
Substance use disorders	0.94	0.60–1.43
Psychosis	2.02	1.59–2.59 ***
Other mental disorders	0.84	0.55–1.26
Suicide attempt		
No	Ref	
Yes	0.26	0.15–0.42 ***
**Contextual characteristics**		
Year		
2002	Ref	
2007	1.61	1.21- 2.16 **
2012	1.73	1.31–2.32 ***
Psychiatric service		
Centro Hospitalar de Lisboa Ocidental EPE	Ref	
Hospital de Magalhães Lemos EPE	0.65	0.49–0.86 **
Centro Hospitalar Psiquiátrico de Lisboa	0.67	0.49–0.90 **
Hospital Professor Doutor Fernando Fonseca EPE	0.79	0.54–1.14
Unidade Local de Saúde do Baixo Alentejo EPE	0.67	0.46–0.96 *

^*^p < 0.05; **p < 0.01; ***p < 0.001

Holding all other variables constant, men have an increment of 1.31 involuntary hospitalizations when compared to women (95%CI 1.06–1.62, p < 0.05). Participants who are married or cohabitating have a 26% decrease in the expected number of involuntary hospitalizations when compared to participants who are single (95%CI 0.56–0.99, p < 0.05). Participants with secondary education and with higher education have 45% and 89% more involuntary hospitalizations than participants with no or primary education, respectively (95%CI 1.05–2.01, p < 0.05, and 95%CI 1.38–2.60, p < 0.001). Participants with a diagnosis of psychosis have an increment of 2.02 involuntary hospitalizations when compared to participants with mood and anxiety disorders (95%CI 1.59–2.59, p < 0.001). Participants with a suicide attempt have a decrease of 74% in the estimated mean number of involuntary hospitalizations when compared to participants with no suicide attempt (95%CI 0.15–0.42, p < 0.001). Participants admitted in 2007 and in 2012 have a 61% and 73% increase in the expected number of involuntary hospitalizations when compared to participants admitted in 2002, respectively (95%CI 1.21–2.16, p < 0.01, and 95%CI 1.31–2.32, p < 0.001). Participants from Hospital de Magalhães Lemos EPE, Centro Hospitalar Psiquiátrico de Lisboa and Unidade Local de Saúde do Baixo Alentejo EPE have a decrease in the expected number of involuntary hospitalizations of 35%, 33% and 33% when compared to participants from Centro Hospitalar de Lisboa Ocidental EPE, respectively (95%CI 0.49–0.86, p < 0.01, 95%CI 0.49–0.90, p < 0.01, and 95%CI 0.46–0.96, p < 0.05).

## Discussion

This study evaluated clinical data from all acute inpatients from five psychiatric departments serving different catchment areas in Portugal in the years of 2002, 2007 and 2012, and identified several sociodemographic, clinical, and contextual factors associated with involuntary psychiatric hospitalizations in Portugal.

Factors that were associated with an increment in involuntary hospitalizations were male gender, secondary or higher education, a psychiatric diagnosis of psychosis, and hospital admission in 2007 and 2012. Factors that were associated with a reduction in involuntary hospitalizations were being married or cohabitating, having experienced a suicide attempt, and belonging to the catchment area of three of the psychiatric services evaluated (the psychiatric hospitals in Lisboa and Porto, and the general hospital in Alentejo).

This research found that people with a psychotic disorder are at higher risk for involuntary hospitalization, one of the most consistent findings from studies around the world [31, 32, 45–62]. It is reassuring that people with the most severe and disabling mental health conditions are also those who most frequently use mental health legislation [4]. Since no definition of diagnosis is provided by legal frameworks, it is important to understand what specific pathways and mechanisms might increase the risk for involuntary admission in someone with psychosis. One study found that hostility and suspiciousness were significant compulsory admission determinants, and that diagnosis no longer had any independent influence on the risk of involuntary hospitalization, after controlling for these specific symptoms [48]. A high level of suspiciousness and uncooperativeness might go hand in hand with reduced coping-strategies and insight, and lead to poor adherence to medication and impaired capacity to establish a therapeutic alliance [51, 60, 62], which explains the higher risk of involuntary hospitalization in psychosis. Another study concluded that aggression and psychotic symptoms increased the odds of involuntary hospitalizations [63]. Increased stress-level and aggressive behaviors might be perceived as an imminent danger to self or others, reflecting the still widespread assumption that people with severe mental disorders are unpredictable and dangerous. This might be a central factor in the judgment of mental health professionals regarding involuntary admission [38]. It is also likely that the shortage of community services for early recognition and assertive outreach is particularly serious in cases of psychosis, leading to a higher rate of acute psychiatric crises and emergency admittances among this group [55].

Regarding sociodemographic factors, male gender was significantly associated with a higher risk of involuntary hospitalizations. This finding is congruent with several previous studies [31, 32, 45–47, 52, 53, 55, 57, 58, 60, 64], while other studies have shown a higher risk in female gender [51, 65, 66]. Possible explanations might be related to societal attitudes and treatment culture that lead to different help-seeking behavior in males and females. Alternatively, mentally ill men may be perceived as more violent, suggesting that perceptions of dangerousness and of overtly dangerous behavior are important contributing factors to involuntary hospitalizations [31, 32, 53, 60]. It is important to know that gender independently influences the risk of involuntary hospitalization. On the one hand, this provides evidence for the possible need to plan mental health services with differing pathways to care for women and men with severe mental disorders. On the other hand, this draws attention to issues relating to equality and to human rights that may be present in mental health legislation, in mental health services, or in potentially discriminatory practices by third parties, as for instance the police [64].

Mixed results have been found regarding the association between educational level and involuntary hospitalization. The finding that a higher educational level is a risk factor for involuntary hospitalizations is in line with some studies [51, 62] but inconsistent with others [53, 58, 67]. Evidence is scarce and difficult to interpret. However, it has been hypothesized that schooling may be associated with greater awareness of individual rights, leading the patient to disagree with inpatient treatment [51].

Regarding marital status, most previous studies have shown that being married is associated with a reduced risk [46, 68] and that being unmarried is associated with a higher risk of involuntary hospitalizations [47, 51, 57, 61, 67]. However, one study showed that married status is associated with an increased risk of involuntary treatment [62]. Overall, the finding of a greater likelihood of involuntary care among unmarried people may reflect the associations between poorer social capability, loneliness, scant social support, and severe mental health difficulties [4, 51, 61]. It might also reflect the role that friends and family may have in encouraging and facilitating help-seeking by voluntary means [4].

In line with some studies [47, 48, 56, 62] but contradicting others [54], we found that a history of suicidal attempt within the previous 12 months was a negative predictor of involuntary treatment. A possible explanation could be that after non-fatal suicidal attempt the individual may receive more social support from family and friends that, in turn, may increase his or her compliance with treatment and hospitalization [47]. Moreover, these patients could gain better insight into the severity of their clinical condition and develop a therapeutic collaboration, learning to ask for help and voluntary hospitalization when in need [48]. Alternatively, individuals with severe physical damage resulting from attempted suicide are voluntarily hospitalized for treatment in general hospitals with consequent referral to psychiatric departments [47].

Previous research suggests several system-related factors to be associated with involuntary hospitalizations: previous utilization of mental health services [53, 69, 70], availability of inpatient beds [34, 52, 71], availability of alternative, less restrictive forms of care, such as temporary housing or residential crisis stabilization [72–74], adequacy of community services [4], availability of home visits [75, 76], lower levels of service integration [62, 77], referral procedures such as contact with police, referral by physicians who did not know the patient or the professional that requires a compulsory admission [63, 65, 67], and longer waiting times for obtaining appropriate mental health care [62, 75]. This study found variation across psychiatric services, suggesting that service organization plays a role in predicting involuntary hospitalizations. However, the analysis did not include service-level variables and it is not possible to ascertain which aspects of mental health care organization are specifically involved.

Another relevant finding was the increase in involuntary hospitalizations in 2007 and 2012 in comparison to 2002. This may correspond to a time trend, following the increasing rates over time in some European countries [4]. The increment in 2012 may also reflect an association between the Great Recession and involuntary hospitalizations in Portugal. During periods of economic recession, it is plausible that several factors will lower the threshold and shape the decision for an involuntary admission, such as family stress, dearth of social associations, social stigma associated with mental health problems, reduced tolerance for persons with mental illness, declining social capital and increased desire for security in society [60, 78–80]. These factors involve a complex interaction between clinical judgement, patient psychopathology, social variables, fulfilment of legal requirements, and local availability of resources.

The results of this study should be interpreted in the light of several limitations. First, the analysis was based on a retrospective observational study of clinical records and we did not have access to information on several factors that might be helpful in explaining the likelihood of involuntary hospitalization, such as symptom severity, level of psychosocial functioning, level of insight, perceived social support or poor adherence to outpatient treatment. Second, the use of routinely collected clinical data may lead to data quality issues, such as the risk of misclassification or of errors in the data registration process. Third, our data do not have repeated measures in each year but may have repeated measures over the three years. For data collection, we obtained the list of hospitalizations for each year and accessed the clinical files for each patient. In case a patient had more than one hospitalization in that year, we only collected information regarding the last hospitalization, indicating the number of previous involuntary hospitalizations. However, during data analysis, it was not possible to identify the patients with hospitalizations in the three years, due to data protection. Fourth, the dataset did not include system or area-related variables that might describe the organizational, environmental or situational factors influencing involuntary hospitalization. Evidence for an association between availability of inpatient beds and involuntary hospitalization is sparse and inconclusive [4]. Mixed results have been found regarding the adequacy of community services and the rate of involuntary hospitalization. Reduced rates of involuntary care were found to be associated with more home visits [76], with the availability of home visits after 10 p.m. [75], and with the availability of alternative less restrictive forms of care [72, 73]. However, community services which were rated more highly by service users were also associated with greater numbers of involuntary admissions [33]. In this study, it was not possible to conduct a retrospective analysis of the different typologies of service organization that could help to clarify the impact of factors such as referral procedures, use of crisis intervention practices, total number of psychiatric beds, availability of adequate housing, social care, and other support services. Regarding area-level variables, evidence suggests that high rates of involuntary hospitalizations are significantly associated with higher area-level deprivation, rates of unemployment, and population density [4]. On a population level, the areas where the hospitals are located are very diverse when it comes to average monthly earnings, unemployment rate and population density (Table 1). These differences may impact involuntary hospitalizations. Fifth, as our objective was to conduct a descriptive study of the factors that influence the number of hospitalizations in general, the authors chose not to study whether or not there was variability between and within hospitals. This may be a subject of further research. Furthermore, whilst stratification of data by year may have allowed for an examination of variation in the number of hospitalizations by year, our objective was to investigate the risk of involuntary hospitalization as compared with the baseline data of 2002 and not to compare the risk factors for involuntary hospitalization in each year. Sixth, patients from Unidade Local de Saúde do Baixo Alentejo EPE were admitted to Centro Hospitalar Psiquiátrico de Lisboa, which makes interpretation of results more complex. Finally, the findings from this study may allow limited comparisons given the marked differences between mental health systems across different countries.

Despite these limitations, this study provided a detailed analysis of all psychiatric admissions under the Mental Health Act over the course of three different years in several psychiatric departments covering catchment areas with distinct geographical and socioeconomic characteristics. This study did not restrict potential risk factors to patient characteristics alone. A future more in-depth analysis of service and area aspects is needed to lead to better predictions and to provide data for services and policies improvement.

## Conclusions

It is increasingly recognized and prioritized that we need a new approach to mental health care that is based on human rights and oriented towards recovery [81]. Reducing the use of compulsory care should be a policy priority. More evidence is needed on how to reduce involuntary hospitalizations in mental health care, while still preserving the right of people with mental health disorders to receive effective treatments when they are less able to express their own will and preferences [6]. Some interventions have shown effectiveness in reducing the risk of compulsory admissions in adults with severe mental illness, when used in the context of existing mental health systems with a community-based organization of mental healthcare [6]. One such intervention is shared decision-making, for instance advance statements and joint crisis plans with indicators for relapse and future treatment preferences. Another effective intervention is integrated care, such as a 24 h crisis resolution team, or an assertive community treatment, or self-management interventions with a relapse prevention element, or psycho-education and monitoring programs [6, 82–89]. Ensuring that these interventions are offered to high-risk patients could significantly reduce the risk of compulsory admissions.

Further research should focus on a better understanding of the risk factors and clinical decision processes that lead to an involuntary hospitalization and its consequences on treatment outcomes. Another focal point should be the development, implementation and evaluation of interventions which prove effective in reducing involuntary hospitalization. This knowledge is essential to inform the development and implementation of targeted strategies to reduce the use of involuntary hospitalization, to ensure equitable access to psychiatric treatment and to reduce health-care inequalities.

## Data Availability

The datasets generated and analysed during the current study are not publicly available, and the authors are not authorized to share the data.

## Abbreviations

UN: United Nations
CRPD: Convention on the Rights of Persons with Disabilities
GLM: Generalized linear model
95% CI: 95% Confidence interval

## References

[1] Lidz CW (1998). Coercion in psychiatric care: what have we learned from research?. J Am Acad Psychiatry Law.

[2] Salize HJ, Dressing H, Peitz M. Compulsory admission and involuntary treatment of mentally ill patients—legislation and practice in EU-Member States. Mannheim: European Comission—Health & Consumer Protection Directorate-General; 2002.

[3] Freeman MC, Kolappa K, Caldas de Almeida JM, Kleinman A, Makhashvili N, Phakathi S (2015). Reversing hard won victories in the name of human rights: a critique of the General Comment on Article 12 of the UN Convention on the Rights of Persons with Disabilities. Lancet Psychiatry.

[4] Walker S, Mackay E, Barnett P, Sheridan Rains L, Leverton M, Dalton-Locke C (2019). Clinical and social factors associated with increased risk for involuntary psychiatric hospitalisation: a systematic review, meta-analysis, and narrative synthesis. Lancet Psychiatry.

[5] de Jong MH, Oorschot M, Kamperman AM, Brussaard PE, Knijff EM, van de Sande R (2017). Crucial factors preceding compulsory psychiatric admission: a qualitative patient-record study. BMC Psychiatry.

[6] Barbui C, Purgato M, Abdulmalik J, Caldas-de-Almeida JM, Eaton J, Gureje O, et al. Efficacy of interventions to reduce coercive treatment in mental health services: umbrella review of randomised evidence. Br J Psychiatry. 2020;1–11. 10.1192/bjp.2020.144. 10.1192/bjp.2020.144 32847633

[7] Katsakou C, Priebe S (2006). Outcomes of involuntary hospital admission—a review. Acta Psychiatr Scand.

[8] Priebe S, Katsakou C, Amos T, Leese M, Morriss R, Rose D (2009). Patients' views and readmissions 1 year after involuntary hospitalisation. Br J Psychiatry.

[9] Kortrijk HE, Staring AB, van Baars AW, Mulder CL (2010). Involuntary admission may support treatment outcome and motivation in patients receiving assertive community treatment. Soc Psychiatry Psychiatr Epidemiol.

[10] Opjordsmoen S, Friis S, Melle I, Haahr U, Johannessen JO, Larsen TK (2010). A 2-year follow-up of involuntary admission's influence upon adherence and outcome in first-episode psychosis. Acta Psychiatr Scand.

[11] Giacco D, Priebe S (2016). Suicidality and hostility following involuntary hospital treatment. PLoS ONE.

[12] Xu Z, Müller M, Lay B, Oexle N, Drack T, Bleiker M (2018). Involuntary hospitalization, stigma stress and suicidality: a longitudinal study. Soc Psychiatry Psychiatr Epidemiol.

[13] Priebe S, Katsakou C, Glöckner M, Dembinskas A, Fiorillo A, Karastergiou A (2010). Patients' views of involuntary hospital admission after 1 and 3 months: prospective study in 11 European countries. Br J Psychiatry.

[14] Sunkel C (2019). The UN Convention: a service user perspective. World Psychiatry.

[15] Nyttingnes O, Ruud T, Rugkåsa J (2016). 'It's unbelievably humiliating'-Patients' expressions of negative effects of coercion in mental health care. Int J Law Psychiatry.

[16] Kallert TW, Glöckner M, Schützwohl M (2008). Involuntary vs. voluntary hospital admission. A systematic literature review on outcome diversity. Eur Arch Psychiatry Clin Neurosci..

[17] Theodoridou A, Schlatter F, Ajdacic V, Rössler W, Jäger M (2012). Therapeutic relationship in the context of perceived coercion in a psychiatric population. Psychiatry Res.

[18] Swartz MS, Swanson JW, Hannon MJ (2003). Does fear of coercion keep people away from mental health treatment? Evidence from a survey of persons with schizophrenia and mental health professionals. Behav Sci Law.

[19] van der Post LF, Peen J, Visch I, Mulder CL, Beekman AT, Dekker JJ (2014). Patient perspectives and the risk of compulsory admission: the Amsterdam Study of Acute Psychiatry V. Int J Soc Psychiatry.

[20] Sashidharan SP, Mezzina R, Puras D (2019). Reducing coercion in mental healthcare. Epidemiol Psychiatr Sci.

[21] Gooding P, McSherry B, Roper C (2020). Preventing and reducing 'coercion' in mental health services: an international scoping review of English-language studies. Acta Psychiatr Scand.

[22] United Nations (2006). Convention on the rights of persons with disabilities.

[23] Szmukler G (2019). "Capacity", "best interests", "will and preferences" and the UN Convention on the Rights of Persons with Disabilities. World Psychiatry.

[24] Committee on the Rights of Persons with Disabilities. General comment no. 1: Article 12: equality before the law; 2014. https://documents-dds-ny.un.org/doc/UNDOC/GEN/G14/031/20/PDF/G1403120.pdf?OpenElement. Accessed 1 Sept 2020.

[25] Appelbaum PS (2019). Saving the UN Convention on the Rights of Persons with Disabilities—from itself. World Psychiatry.

[26] Caldas de Almeida JM (2019). The CRPD Article 12, the limits of reductionist approaches to complex issues and the necessary search for compromise. World Psychiatry.

[27] Galderisi S (2019). The UN Convention on the Rights of Persons with Disabilities: great opportunities and dangerous interpretations. World Psychiatry.

[28] Puras D, Gooding P (2019). Mental health and human rights in the 21st century. World Psychiatry.

[29] Sugiura K, Mahomed F, Saxena S, Patel V (2020). An end to coercion: rights and decision-making in mental health care. Bull World Health Organ.

[30] de Stefano A, Ducci G (2008). Involuntary admission and compulsory treatment in Europe: an overview. Int J Ment Health.

[31] Donisi V, Tedeschi F, Salazzari D, Amaddeo F (2016). Differences in the use of involuntary admission across the Veneto Region: which role for individual and contextual variables?. Epidemiol Psychiatr Sci.

[32] Salize HJ, Dressing H (2004). Epidemiology of involuntary placement of mentally ill people across the European Union. Br J Psychiatry.

[33] Weich S, McBride O, Twigg L, Duncan C, Keown P, Crepaz-Keay D (2017). Variation in compulsory psychiatric inpatient admission in England: a cross-classified, multilevel analysis. Lancet Psychiatry.

[34] Sheridan Rains L, Zenina T, Dias MC, Jones R, Jeffreys S, Branthonne-Foster S (2019). Variations in patterns of involuntary hospitalisation and in legal frameworks: an international comparative study. Lancet Psychiatry.

[35] Kallert TW, Glöckner M, Onchev G, Raboch J, Karastergiou A, Solomon Z (2005). The EUNOMIA project on coercion in psychiatry: study design and preliminary data. World Psychiatry.

[36] Sashidharan SP, Saraceno B (2017). Is psychiatry becoming more coercive?. BMJ.

[37] Mulder CL (2005). Variations in involuntary commitment in the European Union. Br J Psychiatry.

[38] Rössler W (2019). Factors facilitating or preventing compulsory admission in psychiatry. World Psychiatry.

[39] Assembleia da República. Lei nº36/98. Diário da República n.º 169/1998, Série I-A de 1998-07-24; 1998. https://dre.pt/web/guest/legislacao-consolidada/-/lc/116042193/202003011743/73599437/diplomaPagination/diploma/3?did=75115272. Accessed 1 Sept 2020.

[40] Almeida T, Molodynski A (2016). Compulsory admission and involuntary treatment in Portugal. BJPsych Int.

[41] Cintra P, Pessoa Gil N (coords). História dos Serviços de Saúde Mental. Edições Parsifal; 2016.

[42] Comissão Técnica de Acompanhamento da Reforma da Saúde Mental. Relatório de Avaliação do Programa Nacional de Saúde Mental 2007–2016 e propostas prioritárias para a extensão para 2020. Lisboa; 2017.

[43] Caldas de Almeida JM, Mateus P, Xavier M, Tomé G. Towards community-based and socially inclusive mental health care. Análise da situação em Portugal. Joint Action on Mental Health and Well-being; 2015.

[44] R Core Team. R: a language and environment for statistical computing. R Foundation for Statistical Computing; 2019. https://www.R-project.org/. Accessed 15 Apr 2020.

[45] Crisanti AS, Love EJ (2001). Characteristics of psychiatric inpatients detained under civil commitment legislation: a Canadian study. Int J Law Psychiatry.

[46] Hatling T, Krogen T, Ulleberg P (2002). Compulsory admissions to psychiatric hospitals in Norway—international comparisons and regional variations. J Ment Health.

[47] Bauer A, Rosca P, Grinshpoon A, Khawaled R, Mester R, Yoffe R (2007). Trends in involuntary psychiatric hospitalization in Israel 1991–2000. Int J Law Psychiatry.

[48] Montemagni C, Frieri T, Villari V, Rocca P (2012). Compulsory admissions of emergency psychiatric inpatients in Turin: the role of diagnosis. Prog Neuropsychopharmacol Biol Psychiatry.

[49] Myklebust LH, Sørgaard K, Røtvold K, Wynn R (2012). Factors of importance to involuntary admission. Nord J Psychiatry.

[50] Ng XT, Kelly BD (2012). Voluntary and involuntary care: three-year study of demographic and diagnostic admission statistics at an inner-city adult psychiatry unit. Int J Law Psychiatry.

[51] Chang TM, Ferreira LK, Ferreira MP, Hirata ES (2013). Clinical and demographic differences between voluntary and involuntary psychiatric admissions in a university hospital in Brazil. Cad Saude Publica.

[52] Myklebust LH, Sørgaard K, Wynn R (2014). Local psychiatric beds appear to decrease the use of involuntary admission: a case-registry study. BMC Health Serv Res.

[53] Zhou JS, Xiang YT, Zhu XM, Liang W, Li H, Yi J (2015). Voluntary and involuntary psychiatric admissions in China. Psychiatr Serv.

[54] Balducci PM, Bernardini F, Pauselli L, Tortorella A, Compton MT (2017). Correlates of involuntary admission: findings from an Italian inpatient psychiatric unit. Psychiatr Danub.

[55] Hoffmann K, Haussleiter IS, Illes F, Jendreyschak J, Diehl A, Emons (2017). Preventing involuntary admissions: special needs for distinct patient groups. Ann Gen Psychiatry.

[56] Di Lorenzo R, Vecchi L, Artoni C, Mongelli F, Ferri P (2018). Demographic and clinical characteristics of patients involuntarily hospitalized in an Italian psychiatric ward: a 1-year retrospective analysis. Acta Biomed.

[57] Umama-Agada E, Asghar M, Curley A, Gilhooley J, Duffy RM, Kelly BD (2018). Variations in involuntary admission rates at three psychiatry centres in the Dublin Involuntary Admission Study (DIAS): can the differences be explained?. Int J Law Psychiatry.

[58] Wynn R (2018). Involuntary admission in Norwegian adult psychiatric hospitals: a systematic review. Int J Ment Health Syst.

[59] Arnold BD, Moeller J, Hochstrasser L, Schneeberger AR, Borgwardt S, Lang UE (2019). Compulsory admission to psychiatric wards—who is admitted, and who appeals against admission?. Front Psychiatry.

[60] Hotzy F, Hengartner MP, Hoff P, Jaeger M, Theodoridou A (2019). Clinical and socio-demographic characteristics associated with involuntary admissions in Switzerland between 2008 and 2016: an observational cohort study before and after implementation of the new legislation. Eur Psychiatry.

[61] Ma HJ, Xie B, Shao Y, Huang JJ, Xiao ZP (2019). Changing patterns and influencing factors of involuntary admissions following the implementation of China's mental health law: a 4-year longitudinal investigation. Sci Rep.

[62] Schmitz-Buhl M, Gairing SK, Rietz C, Häussermann P, Zielasek J, Gouzoulis-Mayfrank E (2019). A retrospective analysis of determinants of involuntary psychiatric in-patient treatment. BMC Psychiatry.

[63] Silva B, Golay P, Morandi S (2018). Factors associated with involuntary hospitalisation for psychiatric patients in Switzerland: a retrospective study. BMC Psychiatry.

[64] Curley A, Agada E, Emechebe A, Anamdi C, Ng XT, Duffy R (2016). Exploring and explaining involuntary care: the relationship between psychiatric admission status, gender and other demographic and clinical variables. Int J Law Psychiatry.

[65] Eytan A, Chatton A, Safran E, Khazaal Y (2013). Impact of psychiatrists' qualifications on the rate of compulsory admissions. Psychiatr Q.

[66] Indu NV, Vidhukumar K, Sarma PS (2018). Determinants of compulsory admissions in a state psychiatric hospital-Case control study. Asian J Psychiatr.

[67] Hustoft K, Larsen TK, Auestad B, Joa I, Johannessen JO, Ruud T (2013). Predictors of involuntary hospitalizations to acute psychiatry. Int J Law Psychiatry.

[68] Thomsen C, Starkopf L, Hastrup LH, Andersen PK, Nordentoft M, Benros ME (2017). Risk factors of coercion among psychiatric inpatients: a nationwide register-based cohort study. Soc Psychiatry Psychiatr Epidemiol.

[69] Stylianidis S, Peppou LE, Drakonakis N, Douzenis A, Panagou A, Tsikou K (2017). Mental health care in Athens: are compulsory admissions in Greece a one-way road?. Int J Law Psychiatry.

[70] Lebenbaum M, Chiu M, Vigod S, Kurdyak P (2018). Prevalence and predictors of involuntary psychiatric hospital admissions in Ontario, Canada: a population-based linked administrative database study. BJPsych Open.

[71] Lay B, Nordt C, Rössler W (2011). Variation in use of coercive measures in psychiatric hospitals. Eur Psychiatry.

[72] Johnson S, Nolan F, Pilling S, Sandor A, Hoult J, McKenzie N (2005). Randomised controlled trial of acute mental health care by a crisis resolution team: the north Islington crisis study. BMJ.

[73] Lorant V, Depuydt C, Gillain B, Guillet A, Dubois V (2007). Involuntary commitment in psychiatric care: what drives the decision?. Soc Psychiatry Psychiatr Epidemiol.

[74] McGarvey EL, Leon-Verdin M, Wanchek TN, Bonnie RJ (2013). Decisions to initiate involuntary commitment: the role of intensive community services and other factors. Psychiatr Serv.

[75] Bindman J, Tighe J, Thornicroft G, Leese M (2002). Poverty, poor services, and compulsory psychiatric admission in England. Soc Psychiatry Psychiatr Epidemiol.

[76] Emons B, Haussleiter IS, Kalthoff J, Schramm A, Hoffmann K, Jendreyschak J (2014). Impact of social-psychiatric services and psychiatric clinics on involuntary admissions. Int J Soc Psychiatry.

[77] Wierdsma AI, Mulder CL (2009). Does mental health service integration affect compulsory admissions?. Int J Integr Care.

[78] Kessell ER, Catalano RA, Christy A, Monahan J (2006). Rates of unemployment and incidence of police-initiated examinations for involuntary hospitalization in Florida. Psychiatr Serv.

[79] Economou M, Lazaratou H, Ploumpidis D (2018). Compulsory admissions in Greece: multifaceted action is required. Lancet.

[80] Stylianidis S, Souliotis K (2019). The impact of the long-lasting socioeconomic crisis in Greece. BJPsych Int.

[81] Funk M, Drew N (2017). WHO QualityRights: transforming mental health services. Lancet Psychiatry.

[82] Fiorillo A, De Rosa C, Del Vecchio V, Jurjanz L, Schnall K, Onchev G (2011). How to improve clinical practice on involuntary hospital admissions of psychiatric patients: suggestions from the EUNOMIA study. Eur Psychiatry.

[83] de Jong MH, Kamperman AM, Oorschot M, Priebe S, Bramer W, van de Sande R (2016). Interventions to reduce compulsory psychiatric admissions: a systematic review and meta-analysis. JAMA Psychiat.

[84] Aagaard J, Tuszewski B, Kølbæk P (2017). Does assertive community treatment reduce the use of compulsory admissions?. Arch Psychiatr Nurs.

[85] Lay B, Kawohl W, Rössler W (2018). Outcomes of a psycho-education and monitoring programme to prevent compulsory admission to psychiatric inpatient care: a randomised controlled trial. Psychol Med.

[86] Bone JK, McCloud T, Scott HR, Machin K, Markham S, Persaud K (2019). Psychosocial interventions to reduce compulsory psychiatric admissions: a rapid evidence synthesis. EClinicalMedicine.

[87] Molyneaux E, Turner A, Candy B, Landau S, Johnson S, Lloyd-Evans B (2019). Crisis-planning interventions for people with psychotic illness or bipolar disorder: systematic review and meta-analyses. BJPsych Open.

[88] Morán-Sánchez I, Bernal-López MA, Pérez-Cárceles MD (2020). Compulsory admissions and preferences in decision-making in patients with psychotic and bipolar disorders. Soc Psychiatry Psychiatr Epidemiol.

[89] Schöttle D, Ruppelt F, Schimmelmann BG, Karow A, Bussopulos A, Gallinat J (2019). Reduction of involuntary admissions in patients with severe psychotic disorders treated in the ACCESS Integrated Care Model including Therapeutic Assertive Community Treatment. Front Psychiatry.

